# Gender differences in the quantitative and qualitative assessment of chronic pain among older people

**DOI:** 10.3389/fpubh.2024.1344381

**Published:** 2024-06-10

**Authors:** Grażyna Puto, Iwona Repka, Agnieszka Gniadek

**Affiliations:** Faculty of Health Sciences, Institute of Nursing and Midwifery, Jagiellonian University Medical College, Cracow, Poland

**Keywords:** chronic pain, older people, McGill-Melzak pain assessment, gender, gender differences

## Abstract

**Background:**

Pain, regardless of its causes, is a subjective and multidimensional experience that consists of sensory, emotional and cognitive factors that cannot be adequately captured by a single number on a pain scale. The aim of the study was to understand gender differences in the assessment of quantitative and qualitative chronic pain among older people.

**Methods:**

The study used a questionnaire that included questions about demographic and social characteristics as well as the following scales: Abbreviated Mental Score (AMTS), Personal Activities of Daily Living (PADL) by Katz, Instrumental Activities of Daily Living (IADL) by Lawton, Geriatric Depression Scale (GDS-15), McGill Pain Questionnaire (MPQ).

**Results:**

The pain rating index based on rank values of adjectives was higher among women than men (18.36 ± 7.81 vs. 17.17 ± 9.69, *p* = 0.04). The analysis of the frequency of selection of individual adjectives describing the sensory aspects of pain showed that men described the pain as “stabbing” more often than women (26.1% vs. 14.3%, *p* < 0.05). Women chose adjectives from the emotional category more often than men (59.8% vs. 75.4%, *p* < 0.05), describing the pain as “disgusting” (8.9% vs. 1.4%, *p* < 0.05), “unbearable” (19.6 vs. 4.3, *p* < 0.05). In the subjective category, there was a difference between women and men in terms of describing pain as “terrible” (23.2% vs. 7.2%, *p* < 0.05) and as “unpleasant” (11.6% vs. 23.3%, *p* < 0 0.05).

**Conclusion:**

When referring to pain, women tend to employ more detailed and factual language, indicative of heightened emotional sensitivity. Men tend to use fewer words and focus on the sensory aspects of pain. Subjective aspects of pain were demonstrated by both women and men.

## 1 Introduction

The aging population is the fastest growing social group, particularly susceptible to chronic pain (CP) and its adverse consequences (1, 2). This age group is more likely to suffer from diseases that increase the risk of experiencing pain. Pain is not inherently a physiological part of aging, but the aging process of individual systems and organs contributes to its development.

Pain, regardless of its causes, is a subjective and multidimensional experience that consists of sensory, emotional and cognitive factors that cannot be expressed by a single number on a pain scale (2–4). Frequently, especially in older people, there are no changes explaining its development, which requires the use of tools that, apart from intensity, will also assess its other qualitative dimensions. There are many forms of atypical pain in older people, characterized by variability in intensity and location, and the lack of any changes explaining its occurrence (2, 5). Older people often do not use the word “pain” but clearly describe the discomfort or aching they feel. For the initial pain assessment and evaluation of the effectiveness of various interventions, it is important to develop a common language, which is crucial in describing pain in real-life interactions aimed at alleviating the person’s suffering. Pain is a difficult experience to communicate to others and largely depends on the language in which it is to be conveyed. The language used to describe pain is therefore an important aspect of understanding and assessing another person’s pain (6). Pain assessment based on words selected by the subject is considered to be the most illustrative of their current pain experience (2, 7).

A growing body of research shows differences in the pain experienced by women and men. However, there is limited research exploring gender differences in the language used to report pain, considering gender in its biological, psychological, and social dimensions (2, 6, 8–10). Women experience pain more often than men (11–14). Studies show a gender difference in the feeling and perception of pain, which increases with age (4, 14, 15). Women have a lower pain threshold (i.e., the level of perception of a painful stimulus) (13, 16, 17) and tolerance (i.e., the greatest level of pain a person can tolerate) (13, 15, 16), they experience it in more locations (13), with greater frequency and for longer than men (13, 18). Women are more likely than men to suffer from comorbidities (19, 20), worse functional status (20–22), and less physical activity (20, 21), which may contribute to an increased risk of pain (20). Moreover, women seek medical help more often than men because of the pain they experience (23). Differences between men and women also occur in response to pharmacological and non-pharmacological treatments (13, 19, 24–26). Research shows that women use more painkillers (2, 25), demonstrate greater sensitivity to both drug dosage and type (13). Pain management strategies that target functional disability may be particularly important in managing pain in women, who report more pain-related disability than men (22).

The mechanisms underlying the intensity and impact of pain determined by gender differences are not thoroughly understood and require further research to identify potential causes and develop treatment protocols taking into account gender (27–29). The McGill–Melzak Pain Questionnaire (MPQ) allows individuals to provide verbal descriptions that assess both the quantitative and qualitative dimensions of their pain experiences, taking into account the sensory (strength, dynamics and quality of pain), emotional (emotional attitude toward experienced pain) and cognitive (understanding of pain by an older person) aspects (30–32).

There are few reports in the literature on the assessment of pain in older people using the MPQ questionnaire. Most of the research conducted so far is selective and concerns a younger population. Moreover, the MPQ has not yet been used in older adults to compare verbal ratings of chronic pain in men and women (33). Therefore, the purpose of this study was to explore gender differences in the assessment of quantitative and qualitative chronic pain among older people.

## 2 Materials and methods

The research was carried out in medical treatment wards of four hospitals located in the Lesser Poland Voivodeship. After obtaining consent from the management of the institutions where the study was conducted, a pilot study was carried out to verify the tools used. Data were collected in the years 2016–2018 after analyzing medical records. The analysis of medical records was aimed at learning the medical diagnosis and obtaining information regarding the presence of pain, its duration and intensity. The inclusion criteria for the study were: being over 65 years of age, presence of pain for more than 6 months, no diagnosis of cancer, no cognitive impairment (AMTS score > 3), going through a stabilized disease period, obtaining written consent to participate in the study from the subject. The exclusion criteria from the study were: being under 65 years of age, presence of pain for less than 6 months, diagnosed cancer, cognitive impairment (AMTS score < 3), unstable period of the disease, lack of written consent to participate in the study.

After obtaining the patient’s written consent, subjects meeting the inclusion criteria were informed about the purpose of the study, as well as the possibility of asking questions and resigning from participation at any stage. The interview was conducted by a member of the research team at the hospital in conditions convenient for the interviewed person (time, place, in the absence of other people). Each respondent’s task was to choose one answer from the distractors assigned to a given question. The duration of the examination was approximately 15–20 min. Due to the need to access the documentation, the research was not anonymous. The results were encoded, making it impossible to recognize the subject. The collected data was collected in an Excel spreadsheet of the MS Office package and processed using statistical analysis.

The research was carried out as part of the statutory research “Chronic pain in people over 65 years of age” K/ZDS/005733, for which consent was obtained from the Bioethics Committee KBET/83/B/2013 on May 9, 2013.

### 2.1 Statistical analysis

Distributions of qualitative variables were described by the absolute number of cases in individual categories (N) and their percentage share in the distribution of the variable (%). The average values of quantitative variables were described using mean and standard deviation (SD). Relationships between qualitative variables were presented in the form of cross tables. The analysis of statistical significance of these relationships was performed using Pearson’s chi^2^ test. Differences between genders in proportion of selected pain descriptions were computed using *z* test and indicated if p for particular comparison was less than 0.05. Comparison of the average values of quantitative variables with normal distributions were performed using Student *t*-test for independent samples, whereas for distributions significantly different from the normal one with the use the Mann–Whitney test. The strength of relationship between two quantitative variables was estimated using Spearman’s Rho coefficient. Calculations were performed using IBM SPSS Statistics 27 for Windows (IBM Corp. Released 2020. IBM SPSS Statistics for Windows, Version 27.0. Armonk, NY: IBM Corp.).

### 2.2 Instruments

#### 2.2.1 General characteristics

The study used a questionnaire that included questions about demographic and social characteristics (age, gender, education, place of residence, marital status, structure of residence) and an assessment of the clinical condition.

Cognitive function was assessed using the Abbreviated Mental Test Score (AMTS) intended for screening assessment of episodic, semantic and working memory (34). Assessment of functional ability in performing basic activities of daily living (Personal Activities of Daily Living, PADL) was performed using the Katz ADL scale (35), while instrumental activities of daily living (Instrumental Activities of Daily Living, IADL) was performed using the Lawton scale (36). Feelings of depression were assessed using Geriatric Depression Scale (GDS-15) (37, 38).

#### 2.2.2 McGill–Melzak pain questionnaire

Quantitative and qualitative assessment of pain experiences, taking into account their sensory dimension (concerns the strength, dynamics and quality of pain), emotional dimension (includes the emotional attitude toward the pain experience) and cognitive dimension (the patient’s understanding of pain), was carried out using the McGill Pain Questionnaire (MPQ). The multidimensional nature of the questionnaire allows for the assessment of:

1) the location of pain (sensory dimension), in a drawing of the human body with the front and back sides, the study participants indicate the areas of pain. The number of pain sites is summed as an index of the sensory pain dimension;
2) Present Pain Intensity (PPI) is rated on a six-point scale (0 = none, 1 = mild, 2 = discomfort, 3 = anxiety, 4 = terrible and 5 = excruciating), which quantifies pain at the time of examination;
3) the quality of pain (emotional and cognitive dimensions) is assessed using 78 adjectives/words divided into 4 categories and 20 groups. First category describes the sensory dimension of pain (sensory – S 1–10). Second category refers to the affective aspect of pain (affective – A 11–15). The third category includes general assessment of pain as a subjective experience (evaluative – E/OC 16). The fourth category (items 17–20) verifies various aspects of pain. It consists of three groups of words describing the miscellaneous sensory properties [(M(S) 17–19] and the miscellaneous affective and evaluative [M(AE) 20] aspects. Within each category, adjectives were grouped in order from the weakest to the strongest pain intensity. The subjects chose the words (after reading the entire list to become familiar with the terms) that illustrate current pain sensations, with the condition that they can only choose one word from each group. Thus, the results of the questionnaire enable obtaining measurement data:

- number of word chosen (NWC) where as the number of words chosen increases, the intensity of pain increases;
- Pain Rating Index (PRI) based on rank values of adjectives in groups, where the position of the adjective corresponds to its value placed on the list. Those values are summed to obtain the Pain Rating Index-Total [PRI (T)] which is the average of the number of selected words. Points are awarded to individual words, depending on their position in a given subgroup – the first word receives the value “1,” the second “2” (at the end, the values of individual words are added up).

The questionnaire contains additional items regarding: accompanying symptoms (vomiting, headaches, dizziness, drowsiness, constipation, diarrhea), nutrition and appetite (good, reduced, limited), characteristics of pain (continuous, intermittent, paroxysmal), sleep (good, intermittent, insomnia), and activities (full, limited) (30–32, 39).

## 3 Results

### 3.1 Resource identification initiative demographic, social and clinical characteristics

Among 181 people (112 women, 69 men) over 65 years of age included in the presented analysis, the percentage of surveyed women was higher compared to the percentage of surveyed men (61% vs. 38%). The average age of the surveyed women was 77.3 years (±8.0) and the average age of men was 76.7 years (±7.7). There were no statistically significant differences (Table 1).

**Table 1:** Socio-demographic and clinical characteristics.

Socio-demographic characteristics	Women *N* = 112 (61.9%)	Men *N* = 69 (38.1%)	*p*
	Mean ± SD	Mean ± SD	
Age (years)	77.3 ± 8.0	76.7 ± 7.7	0.69
Education	*N* (%)	*N* (%)	
Basic	11 (9.8)	9 (13.0)	0.02
Vocational	18 (16.1)	18 (26.1)	
Secondary	63 (56.3)	23 (33.3)	
Higher	20 (17.9)	19 (27.5)	
**Place of residence**			
City	82 (73.2)	45 (65.2)	0.253
Countryside	30 (26.8)	24 (34.8)	
**Marital status**			
Married	41 (36.6)	40 (58.0)	0.00
Divorced or widowed	71 (63.4)	29 (42.0)	
**Financial situation**			
Gets by	63 (56.3)	37 (53.6)	0.730
Is not enough or barely enough to make ends meet	49 (43.8)	32 (46.4)	
**Residence structure**			
Living alone	24 (21.4)	12 (17.4)	0.252
With a partner	18 (16.1)	18 (26.1)	
With the family	70 (62.5)	39 (56.5)	
Clinical characteristics	Mean ± SD	Mean ± SD	
Number of comorbidities	3.98 ± 2.27	4.38 ± 2.67	0.31
Geriatric depression scale (range 0–15)	6.92 ± 2.75	6.42 ± 2.87	0.24
AMTS (range 4–10)	8.29 ± 1.64	8.65 ± 1.47	0.15
ADL Katz scale (range 0–6)	4.73 ± 1.59	5.55 ± 0.89	<0.001
Lawton’s IADL scale (range 0–27)	18.28 ± 4.78	20.53 ± 4.75	0.002

N, number of respondents; %, percentage of respondents; SD, standard deviation; p, value; ^M-W^ for the test Mann–Whitney p; ^S^ for Student’s *t* test; NS, statistically insignificant.

### 3.2 Pain characteristics

The most common places of pain among both women and men were the lower limbs (47.8% vs. 52.7%) and the lumbar-sacral area (40.2% vs. 33.3%). Both women and men most often indicated 1 place of pain; 3 places and more were indicated more often by women than men. There were no significant relationships between the number of pain sites and the gender of the examined subjects (Table 2).

**Table 2:** Location of pain sites in the study group.

Location of pain areas		Women *N* (%)	Men *N* (%)	*p*
Location of pain	Head	8 (7.1)	5 (7.2)	0.98
	Belly	21 (18.8)	17 (24.6)	0.34
	Back/lumbar sacral area	45 (40.2)	23 (33.3)	0.36
	Generalized pain throughout the body	19 (17.0)	7 (10.1)	0.20
	Mediastinum	12 (10.7)	7 (10.1)	0.90
	Upper limbs	20 (17.9)	15 (21.7)	0.52
	Lower limbs	107 (47.8)	59 (52.7)	0.53
Number of pain spots	1 spot	42 (37.5)	29 (42.0)	0.72
	2 spots	33 (29.5)	21 (30.4)	
	3 spots and more	37 (33.0)	19 (27.5)	

*(N) the number does not add up to 100% because the respondents could indicate many places of pain and could report pain in several parts of the body.

### 3.3 Quantitative and qualitative assessment of pain sensations in the study group

Present Pain Intensity (PPI) expressing the quantitative assessment of pain at the time of examination did not show significant differences between women and men—the average pain intensity was higher among women than among men (3.29 ± 0.61 vs. 3.23 ± 0.62). The pain rating index (PRI) based on rank values of adjectives was significantly higher among women than men (18.36 ± 7.81 vs. 17.17 ± 9.69, *p* = 0.04).

The qualitative assessment of pain experiences showed that the average miscellaneous affective/evaluative (M(AE) 20) category was significantly higher among women than men (1.44 ± 1.30 vs. 0.84 ± 0.99, *p* = 0.001) (Table 3).

**Table 3:** Pain components according to the McGill–Melzack questionnaire.

Pain components according to the MPQ scale		Women Mean ± SD	Men Mean ± SD	*p*
McGill Pain Questionnaire (MPQ)	Sensory (S 1–10)	7.50 ± 5.65	6.23 ± 8.19	0.44
	Affective (A 11–15)	1.94 ± 1.80	1.72 ± 2.31	0.06
	Evaluative (E 16)	2/05 ± 1/41	1.85 ± 1.31	0.33
	Miscellaneous sensory (M(S) 17–19)	1.99 ± 1.88	1.87 ± 1.65	0.83
	Miscellaneous affective/evaluative (M(AE) 20)	1.44 ± 1.30	0.84 ± 0.99	0.001
	Present Pain Intensity (PPI)	3.29 ± 0.61	3.23 ± 0.62	0.56
	Number of words chosen (NWC)	7.75 ± 3.87	7.51 ± 3.98	0.28
	Pain Rating Index – Total [PRI (T)]	18.36 ± 7.81	17.17 ± 9.69	0.04
	Pain Rating Index (PRI) based on rank values	2.43 ± 0.57	2.29 ± 0.44	0.06
		*N* (%)	*N* (%)	0.41
Subjective assessment of nutritional status	Good	78 (69.6)	42 (60.9)	
	Reduced	26 (23.2)	19 (27.5)	
	Limited	8 (7.1)	8 (11.6)	
The nature of the pain	Continuous	25 (22.3)	12 (17.4)	0.58
	Intermittent	40 (35.7)	23 (33.3)	
	Paroxysmal	47 (42.0)	34 (49.3)	
The nature of the sleep	Good	6 (5.4)	9 (13.0)	0.06
	Intermittent	84 (75.0)	53 (76.8)	
	Insomnia	22 (19.6)	7 (10.1)	
Daily activity	Full	25 (22.3)	14 (20.3)	0.75
	Limited	87 (77.7)	55 (79.7)	
Accompanying symptoms	Vomiting	11 (9.8)	7 (10.1)	0.94
	Headaches	19 (17.0)	7 (10.1)	0.20
	Dizziness	37 (33.0)	20 (29.0)	0.57
	Somnolence	5 (4.5)	0 (0.0)	0.16
	Constipation	22 (19.6)	12 (17.4)	0.71
	Diarrhea	9 (8.0)	10 (14.5)	0.17

*(N) number of respondents confirming symptoms.

### 3.4 Gender differences in the number and frequency of words selected

The analysis of the frequency of selection of individual adjectives describing the sensory aspects of pain (S 1–10) showed that men described their pain as “stabbing” significantly more often than women (26.1% vs. 14.3%, *p* < 0.05). From the affective category (A11–15), women chose adjectives significantly more often than men (59.8% vs. 75.4%, *p* < 0.05), while in the evaluative category, a significant difference was found between women and men in terms of defining pain as “terrible” (23.2% vs. 7.2%, *p* < 0.05), and as “unpleasant” (11.6% vs. 23.3%, *p* < 0.05). In the group miscellaneous evaluative/affective (M(AE) 20), women significantly more often than men chose the adjective “disgusting” (8.9% vs. 1.4%, *p* < 0.05), “unbearable” (19.6 vs. 4.3, *p* < 0 0.05), while men—significantly more often than women—did not choose adjectives from this group (20.5% vs. 37.7%, *p* < 0.05).

### 3.5 Factors correlating with pain in the study group

Spearman’s Rho coefficient in the studied group of women showed a significantly negative correlation between:

- the sensory dimension of pain and cognitive functions (with the increase in the sensory dimension of pain, the efficiency of cognitive functions decreased);
- subjective aspects of pain and basic activities of daily living (as the sensation of pain increased, the ability to perform basic activities of daily living decreased);
- subjective aspects of pain and the severity of depression symptoms (as the sensation of pain increased, the feeling of depression decreased);
- emotional aspects of pain and basic activities of daily living (as the emotional aspects of pain increased, the ability to perform basic activities of daily living decreased);
- present pain intensity and cognitive functions (as the present pain intensity increased, cognitive functions decreased);
- present pain intensity and basic activities of daily living (as the present intensity of pain increased, the ability to perform basic daily activities decreased);
- present pain intensity and instrumental activities of daily living (as the present intensity of pain increased, the ability to perform instrumental activities of daily living decreased);
- pain rating index based on rank values and basic activities of daily living (as the pain rating index based on rank values increased, the efficiency in basic activities of daily living decreased);
- pain rating index based on rank values and instrumental activities of daily living (as the pain rating index based on rank values increased, the efficiency in instrumental activities of daily living decreased).

Significantly positive correlation in the studied group of women was demonstrated between:

- mixed aspects of pain and basic activities of daily living (as the mixed aspects of pain increased, the ability to perform basic activities of daily living increased);
- mixed aspects of pain and instrumental activities of daily living (as the mixed aspects of pain increased, the ability to perform instrumental activities of daily living increased);
- present pain intensity and the intensity of depression (with the increase in the present intensity of pain, the intensity of the feeling of depression increased).

Spearman’s Rho coefficient in the studied group of men showed a significantly negative correlation between the present pain intensity and cognitive functions (with the increase in pain intensity, cognitive function decreased) (Table 4).

**Table 4:** Spearman’s Rho correlation between pain sensation according to MPQ and AMTS, PADL, IADL, GDS among the subjects.

McGill Pain Questionnaire (MPQ)		Women				Men			
		AMTS	PADL	IADL	GDS	AMTS	PADL	IADL	GDS
Sensory (S 1–10)	rho	−0.19*	0.09	0.02	−0.02	−0.14	−0.01	0.02	0.04
	*p*	0.04	0.36	0.81	0.86	0.24	0.91	0.89	0.76
Affective (A 11–15)	rho	−0.06	−0.01	0.01	0.07	−0.05	0.14	0.12	0.02
	*p*	0.51	0.90	0.93	0.46	0.65	0.25	0.33	0.84
Evaluative (E 16)	rho	−0.003	−0.23*	−0.18	−0.23*	0.11	−0.02	0.05	0.02
	*p*	0.98	0.02	0.06	0.02	0.38	0.87	0.68	0.89
Miscellaneous sensory [M(S) 17–19]	rho	0.06	0.20*	0.21*	−0.11	−0.02	0.06	0.01	−0.11
	*p*	0.50	0.03	0.03	0.27	0.89	0.61	0.92	0.35
Miscellaneous affective/evaluative (M(AE) 20)	rho	−0.03	−0.22*	−0.12	−0.02	0.06	−0.00	−0.01	−0.04
	*p*	0.77	0.02	0.20	0.79	0.65	0.97	0.92	0.74
Present Pain Intensity (PPI)	rho	−0.22*	−0.22*	−0.27**	0.19*	−0.35**	−0.02	−0.05	0.08
	*p*	0.02	0.02	0.004	0.04	0.004	0.86	0.68	0.51
Number of words chosen (NWC)	rho	−0.06	0.15	0.14	0.12	−0.05	0.07	0.10	0.11
	*p*	0.50	0.12	0.13	0.20	0.68	0.56	0.39	0.35
Pain Rating Index – Total [PRI (T)]	rho	−0.12	−0.05	−0.002	0.05	−0.01	−0.02	0.05	0.04
	*p*	0.19	0.63	0.98	0.62	0.92	0.85	0.69	0.73
Pain Rating Index (PRI) based on rank values	rho	−0.03	−0.41**	−0.34**	−0.06	−0.03	−0.20	−0.11	−0.09
	*p*	0.76	0.000	0.000	0.53	0.78	0.10	0.35	0.47

## 4 Discussion

The paper presents gender differences in the quantitative and qualitative assessment of chronic pain in older people using the McGill–Melzak Pain Questionnaire (MPQ). There are few reports in the literature on gender differences in quantitative and qualitative assessment of chronic pain in older people using the MPQ questionnaire. This kind of assessment with the use of the aforementioned diagnostic tools has been performed only occasionally. This is probably due to the fact that it is the physical aspect of pain that is most often used for clinical purposes. The language used by people experiencing pain is much less frequently assessed. The type of language that women and men use when feeling pain is the only source of information about their pain, which has implications for treatment. This study therefore examines the little understood role of language in the description of pain experienced by women and men in real interactions with chronic diseases. In the study, women showed higher emotional sensitivity than men. Women tend to focus on the emotional aspects of the pain they experience: they describe the pain as “disgusting,” “unbearable” and subjectively as “terrible.” Men tend to focus on the sensory aspects, describing the pain as “stabbing,” and its subjective aspects, describing the pain as “unpleasant.” The study conducted by Jaworska and Ryan (27) on spontaneously used adjectives describing the experienced pain in chronic and terminal diseases showed quantitative and qualitative differences between women and men in the ways in which they report pain, indicating the existence of characteristic feminine and masculine adjectives describing pain. While these adjectives conform to some of society’s dominant stereotypes of femininity and masculinity, they also transcend them. Women, as shown in this study, use a wider range of adjectives, referring to the experience with specific and factual terms, as well as cognitive and psychological kinds of vocabulary. The authors of the study explain this difference by the fact that ailments characterized by pain, especially chronic pain, are more common in women, and therefore women can acquire a more diverse vocabulary to talk about pain. In contrast, men tend to use fewer adjectives, most of which are highly emotional. Men most often used words such as “excruciating,” “terrible,” and “bad,” which were not found in women’s narratives. The expression of pain in men’s narratives suggests that they report pain when it results in a feeling of defenselessness and helplessness (27).

Strong et al. (6) found that women used more vivid and sensory language (e.g., “throbbing,” “sharp,” “stabbing”), as well as similes in comparison to men when describing their pain. Women in this study used more words than men when describing their pain experiences (6), which was also confirmed in this study.

The language used to describe pain may differ between men and women experiencing pain because they may have learned different words to describe pain from previous pain experiences, and research has shown that gender differences significantly influence the perception of clinical pain (29). Bartley, in a review of clinical and experimental results, showed that women, in response to a pain stimulus, engage in catastrophizing (i.e., exaggerating the intensity of the experienced pain) (13).

Although it is recognized that pain is common in older people, different criteria for diagnosing pain, the scales used to assess pain intensity, the populations studied (residents of their own homes, long-term care facilities) and the methods used (questionnaires and medical surveys) make it difficult to compare studies and determine the final incidence. Studies consistently show that the incidence of pain increases with age and is higher among women than men (24). In our study, which included people over 60 years of age with chronic pain, the percentage of surveyed women was higher than men (61.9% vs. 38.1%). The study did not analyze the increase in the incidence of pain with age, but it confirmed a higher pain assessment rate in women than in men. As shown in a study by Jiménez-Trujillo et al. (12) higher pain intensity in women than in men resulted in taking more painkillers (12). The most common location of pain among both women and men in the study was the lower limbs and the lumbar-sacral area. Similarly, in a study conducted in the United States, the incidence of knee and lumbar pain was higher in women than in men. This study suggested that women are more prone to pain than men (40). Also, the dominant type of chronic pain in the Finnish aging population was the lower limbs (40%) and thus was reported almost twice as often as the lumbosacral region (21%) (41) In turn, in the nationwide PolSenior study (42) and in the ASPREE study, the most common location of pain was lower back pain, followed by lower limb pain among both women and men (43). Differences between women and men in the occurrence of pain sites have been confirmed in many studies. It has been shown that as the number of reported pain sites increased, the predominance of women over men in the studied populations increased (42, 44). In the present study, every third woman (33%) reported pain in three places and every third man in two places (30.4%). This information should be taken into account when assessing pain, especially in people who verbalize their experiences of pain differently. Understanding the language used by older women and men is important because it will guide the choice of appropriate treatment.

The relationship between chronic pain and functional capacity among both women and men has been confirmed in many studies (21, 45, 46) and the number of pain spots has been indicated as a prognostic factor for functional disability (47, 48). The study also confirmed that as the present pain intensity increased, disability increased in both basic and instrumental everyday activities among women.

Research confirms that the intensity of pain causes an increase in disability. A study conducted in Poland on a representative population of people aged 60 and over showed that an increase of 1 point in the intensity of pain assessed on the Visual Analogue Scale (VAS), resulted in a 27% increase in both PADL and IADL disability. Moreover, this study confirms that pain is strongly related to the fitness of older people, causing disability in a short time (49).

Anxiety and depressive disorders, which are more common among women (20), have been shown in studies to be associated with greater sensitivity and poorer adaptation to pain (20, 50, 51). The study confirmed that as the intensity of pain increased, cognitive performance decreased among both women and men, while the feeling of depression increased only among women. Pain and depression are more common in women than in men and have a bidirectional relationship. Depression and pain may be risk factors for each other (52). This association highlights the complexity of older adults’ experience of pain and the need for a biopsychosocial approach to pain management. Therefore, the assessment of pain in older people is a serious challenge not only because of the possible increase in cognitive impairments and sensory disorders, but also because of insufficient verbalization and reporting of pain by older people who believe that pain is “just a normal part of getting older.”

The present study should be interpreted with several limitations. First, the study was conducted in a clinical setting. The older people participating in the study were patients who were admitted to hospital for various reasons. However, the stay of these people in the hospital allowed for the selection of a group for the study, e.g., the exclusion of people with cancer pain. Secondly, the questionnaire lacks questions on important variables such as the duration of pain-related disability. Even taking into account its limitations, the study makes an important contribution to the current literature for the following reasons. To the best of our knowledge, this is the first quantitative and qualitative study examining pain in older adults. The study analyzed quantitative and qualitative data to better understand the perceived impact of pain, and our qualitative data included closed-ended questions promoting clarity in communication. Often considered time-consuming, the McGill–Melzak Questionnaire helps develop a common language regarding the gaps between patients’ pain expressions and healthcare professionals’ perceptions of pain. Spontaneously uttered simple vocabulary seems problematic because it may not clearly signal the pain experienced by older people.

Further research is needed to elucidate the mechanisms underlying gender differences in responses to chronic pain. Future quantitative and qualitative pain assessment studies among women and men should assess pain experiences determined not only by changing biological but also psychosocial factors that occur during the aging process. Psychosocial factors that are thought to mediate the effects of pain, such as stereotypes, cultural differences, pain-related beliefs, being able to cope, and a sense of self-efficacy may explain the observed differences in pain between women and men.

## 5 Conclusion

In summary, the study identified quantitative and qualitative gender differences in the assessment of chronic pain. When referring to pain, women use a greater number of specific and factual words, demonstrating emotional sensitivity (describing the pain as “disgusting,” “unbearable”). Men tend to use fewer words and focus on the sensory aspects of pain (describing the pain as “stabbing”). Subjective aspects of pain were demonstrated by both women (describing it as “terrible”) and men (describing it as “unpleasant”). Understanding the role of gender in the quantitative and qualitative assessment of pain expressed through language may not only help healthcare professionals respond effectively when talking about pain but also develop more holistic pain assessment and treatment practices.

## Data availability statement

The original contributions presented in the study are included in the article/supplementary material, further inquiries can be directed to the corresponding author.

## Ethics statement

The studies involving humans were approved by The consent of the Bioethics Committee KBET/83/B/2013 of May 9, 2013 was obtained. The studies were conducted in accordance with the local legislation and institutional requirements. The participants provided their written informed consent to participate in this study.

## Author contributions

GP: Conceptualization, Data curation, Formal analysis, Funding acquisition, Investigation, Methodology, Project administration, Resources, Software, Supervision, Visualization, Writing – review & editing. IR: Conceptualization, Formal analysis, Project administration, Writing – review & editing. AG: Formal analysis, Supervision, Visualization, Writing – review & editing.
